# International Group for Research in the Immunopathogenesis of Genital Infections: I. International Symposum on Immunopathogenesis of Chlamydia Infections

**DOI:** 10.1155/S1064744996000257

**Published:** 1996

**Authors:** Steven S. Witkin

**Affiliations:** New York New York USA


					Infectious Diseases in Obstetrics and Gynecology 4:11 I-II5 (1996)
(C) 1996 Wiley-Liss, Inc.
International Group for Research in
the Immunopathogenesis ofGenital
Infections
Sy
I. International mposum on
Immunopathogenesis ofChlamydia Infections
December 6-8, 1996
Cornell University Medical College
1300 York Avenue, New York, NY
USA
Scientific Program Committee
Steven S. Witkin, Ph.D, New York, New York, USA
Sandra Mazzoli, PhD, Florence, Italy
MyriamAskeinazy-Elbhar, PhD, Paris, France
Jeanne Orfila, PhD, Amiens, France
Sponsors
Savyon Diagnostics, Ltd
Pfizer Inc.
Genzyme
Roche Molecular Systems
Department ofObstetrics and Gynecology, Comell University Medical College
Program
Received September 15, 1996
Accepted October I, 1996
INTERNATIONAL SYMPOSIUM: IMMUNOPATHOGENESIS OF CHLAMYDIA INFECTIONS PROGRAM
SYMPOSIUM SCHEDULE
FRIDAY, DECEMBER 6, 1996
Openingreception and complementarybuffet forparticipants and guests, Griffis Faculty Club, Comell
University Medical College, 1300 York Avenue, New York 6:00 -8:00 PM
SATURDAY, DECEMBER 7, 1996
9:00 9:10 Welcome
9:10 9:20 Greeting
Steven Witkin
William J. Ledger
9:20- 9:50
9:50 10:20
10:20 10:50
10:50 11:00
11:00- 11:10
SESSION I. WILLIAM J. LEDGER, CHAIRPERSON
Immune Responses to Intracellular Pathogens
Mario Clerici, Milan, Italy
Immunology ofNon-trachomatis Chlamydial Infections
Jeanne Orfila, Amiens, France
Immunopathogenesis of Chlamydiapneumoniae Infections in Children
Margaret Hammerschlag, Brooklyn, New York, USA
Cytokine Profile ofChlamydiapneumoniae Infected U-937 Macrophages
C. A. Gaydos, D.G. Pham, L. Bobo, T.C. Quinn, Baltimore, Maryland, USA
Production oflL-4 and IL-6 in HEp 2 Cells Infected with Chlamydiapneumoniae
Patricia M. Roblin, A. Kutlin, M.R. Hammerschlag, Brooklyn, New York, USA
11:10 11:30 COFFEE BREAK
11:30 11:40
11:40 -11:50
11:50 12:00
12:00 12:10
12:10 12:20
12:20- 12:30
12:30- 12:40
Chlamydiapneumoniae Induced Atherosclerosis in Rabbits
K. Laitinen, P. Saikku, Oulu, Finland
SeroCP -A Serological Assay for the Detection of Chlamydiapneumoniae IgG,
IgA and IgM Antibodies
A. Groh, V. Prankkratova, M. Lipson, A. Ben-Michael, O. Horovitz, R.
Moschkovitz, Jena, Germany
Protective Immunity Against Chlamydiapsittaci Serotype Strains
Carlos De Sa, J. Salinas, F. Bernard, A. Souriau, A. Rodolakis,Nouzilly, France
Repetitive Extragenic Palindromic Sequence Polymorphism Based Polymerase
Chain Reaction (REP-PCR) is a Useful Tool in Diagnostics ofChlamydiapsittaci
Infection in Sheep
S.P. Martinov. E. Tchemeva, B. Jersek, Sofia, Bulgaria
Human Studies on the Association ofCellular Immune Factors in Trachoma with
Infection and Diseases
L.D. Bob0, N.G. Novak, S. Vitale, H. Mkocha, Y.H. Hsieh, B. Munoz, S. West,
T.C. Quinn, Baltimore, Maryland, USA
Scarring Trachoma is Associatedwith Polymorphism in the TNF-alpha Gene Pro-
moter and with Elevated TNF-alpha in Tear Fluid
D.J. Conway, M.J. Holland, R.L. Bailey, A.E. Campbell, S. Mahdi, R. Jennings,
E. Mbena, David Mbey, London, United Kingdom
Characterization ofthe Local Immune Response to Chlamydiatrachomatis among
INFECTIOUSDISEASES IN OBSTETMCSAND GYNECOLOGY
INTERNATIONAL SYMPOSIUM: IMMUNOPATHOGENESIS OF CHLAMYDIA INFECTIONS PROGRAM
Trachoma Patients with Conjuctival Scarring
Stephanie Phan. R. Pham, D. Dean, San Francisco, California, USA
12:40- 2:40 LUNCH
SESSION II. GERRY BYRNE, CHAIRPERSON
2:40- 3:10
3"10- 3:40
3:40- 3:50
3:50 4:00
4:00-4"10
4:10-4:40
4:40-4:50
4:50 -5:00
5:00- 5"10
Immune Responses in the Male Genital Tract
Steven Witkin, New York, New York, USA
Clinical Consequences ofImmune Responses to Chlamydia in Men
Sandra Mazzoli, Florence, Italy
Antigen and Isotype Specificity of Human B Cell Responses in
Immunopathogenesis ofGenital Chlamydia trachomatis Infection,
S. Ghaem-Maghmi, P.E. Hay, D.J.M. Lewis London, United Kingdom
The Relationship Between the 60kD Heat Shock Protein, Antisperm Antibodies
and Undetected Chlamydia trachomatis Infection in Male Partners of Couples
with Undiagnosed Infertility
M. Gladys Munoz. J. Jeremias, S.S. Witkin, Caracas, Venezuela
Chlamydia trachomatis Specific Immunoparameters and Cytokine Production in
Semen from Males Affected by Prostatitis
F... Mea.oci, A. Salvi, S. Mazzoli, Florence, Italy
Immune Responses to Chlamydia trachomatis and Pregnancy and In Vitro Fertili-
zation Outcome
Myriam Askienazy-Elbhar, Paris, France
Detection ofChlamydia trachomatis DNA and Chlamydial Inclusions Using Non-
radioactive in situ Hybridization and Apaap Staining ofPlacental Samples
Mesut Gencay, M. Puolakkainen, T. Wahlstrom, P. Ammala, L. Mannonen, A.
Vaheri, M. Koshiniemi, Helsinki, Finland
Asymptomatic Chlamydia trachomatis Infection and Cervical Cytokines in Women
Undergoing In Vitro Fertilization (IVF)
Myriam Askienazy-Elbhar. F. Olivennes, U. Lundqvist, R. Fanchin, A. Neuer, R.
Frydman, S.S. Witkin, Paris, France
Prevalence of Chlamydia trachomatis Antibodies in Cord Blood
Slobodanka Djukic, M. Nedeljkovic, M. Pervulov, S. Vesic, A. Ljubic, B. Vranes,
Belgrade, Yugoslavia
SUNDAY, DECEMBER 8, 1996
SESSION III. JORMA PAAVONEN, CHAIRPERSON
9:00- 9:30
9:30- 9:40
9:40- 9:50
9:50 10:00
Persistent Chlamydial Infections
Gerry Byme, Madison, Wisconsin, USA
Persistence of Chlamydia trachomatis and Chlamydial Antigens in HEP-2 Cells
and THP-1 Cells after Treatment with Ciprofloxacin
U..Dreses-Werringloer, B. Jurgens, H. Zeidler, S. Ziesing, L. Kohler, Hannover,
Germany
Prevalence and Viability of Chlamydia trachomatis in Infertile Women
H.C. Gerard, P.J. Branigan, S.S. Minassian, A.P. Hudson, Philadelphia, Pennsyl-
vania, USA
Failure to Detect C. trachomatis by MOMP PCR, in Contrast to Cultures with
INFECTIOUSDISEASES IN OBSTETRICSAND GYNECOLOGY 113
INTERNATIONAL SYMPOSIUM: IMMUNOPATHOGENESIS OF CHLAMYDIA INFECTIONS PROGRAM
10:00- 10:10
10"10- 10:40
Multiple Passages, In Situ Hybridization and Immunoperoxidase Staining, in Pel-
vic Samples ofWomen with Tubal Factor Infertility
Jeanine Henry-SuCh, M. Askienazy-Elbhar, J. Orfila, M. Thomas, JF Lefebre, E
Eb, DL Patton, LA Campbell, Paris, France
Viability of Non-Cultivatable Chlamydia trachomatis Persisting in Human Pe-
ripheral Blood Monocytes and Human Monocytic THP-1 Cells
L. Koehler, E. Nettelnbreker, A. Hudson, H. Gerard, C. Bonk, H, Zidlcr,
Hannover, Germany
Heat Shock Proteins and Infection
Steven Witkin, New York, New York, USA
10:40 11"10
11"10- 11:20
11:20 11:30
11:30 11:40
11:40 11:50
11:50 12:00
12:00- 12:10
COFFEE BREAK
Antibodies to Human 60kDa Heat Shock Protein(hsp60) in Sera of Women with
Pelvic Inflammatory Disease Correlate with Immune Response to Conserved B-
Cell Epitope ofthe Chlamydia trachomatis hsp60
K. Bergstrom, S.S. Witkin, J. Paavonen, P-A Mardh, M, D0miko, Uppsala, Swe-
den
Humoral Immunological Response to a Sequence ofthe 57kD Heat Shock Protein
Commonto Mouse and Chlamydiatrachomatis, inMice Inoculatedwith a Chlamy-
dia trachomatis E Strain
M.J. Borrego, V. Fuentes, E. Bissac, M.A. Catry, F. Eb, J. Orfila Lisboa, Portu-
gal
The Presence ofSerum Antibodyto the Chlamydial Heat Shock Protein (CHSP60)
Paul Claman, L Honey, R Peeling, P Jessamine, B Toye, Ottawa, Canada
Distribution ofHeat Shock Proteins in First Trimester Human Decidua
Andreas Neor, P. Ruck, K. Marzusch, J. Diet, E.
Kaiserling, H.P. Horny, S.S. Witkin, Tubingen, Germany
Circulating IgG antibodies to C. trachomatis and the chlamydial, E. coli and hu-
man 60kD heat shock proteins in women
S,S. Witkin, M. Askienazy-Elbar, J. Henry-Suchet, J. Tort-Grumbach, K. Sarjdine,
Paris, France and New York, NY
Serum Antibodies to a Conserved B-Cell Epitope ofChlamydial Heat Shock Pro-
tein 60 in Patients with Sexually Acquired Reactive Arthritis (SARA)
J Domeika, SS Witkin, P-A Mardh, Marius Domeika, Uppsala, Sweden and New
York, New York
12:10- 2:00
SESSION IV.
2:00 2:30
2:30 3:00
3:00 3:30
LUNCH
ROSANNA PEELING, CHAIRPERSON
Immune Consequences of Chlamydia trachomatis Infection in Animal Models
Dorothy Patton, Seattle, Washington, USA
Immune Protection against Chlamydia trachomatis in Females
Richard Morrison, Birmingham, Alabama, USA
Clinical Consequences of Immune Responses to Chlamydia trachomatis Upper
Genital Tract Infections in Women
Jeanine Henry-Suchet, Paris, France
114 INFECTIOUSDISEASESIN OBSTETRICSAND GYNECOLOGY
INTERNATIONAL SYMPOSIUM: IMMUNOPATHOGENESIS OF CHLAMYDIA INFECTIONS PROGRAM
3:30 3:40
3:40- 3:50
3:50 -4:00
4:00 -4:10
4:10 -4:20
Functional Damage ofthe Fallopian Tubes and Immune Responses to Chlamydia
trachomatis
Istvan Sziller, S.S. Witkin, M. Ziegert, P. Fedorcsak, Z. Papp, Budapest, Hungary
Production of Interleukin beta in Response to Chlamydial Infection in an in
vitro Model ofthe Human Fallopian Tube
Kevin Ault, O. Tawfik, M.M. Smith King, J. Gunter, Iowa City, Iowa, USA
SerCTTM A New Peptide Based ELISA Test for Specific Detection ofChlamydia
trachomatis Antibodies
Bells Ohana, V. Prankratova, A. Groh, E. Straube, R. Brand, A. Blaicher, I.
Mezamer-Tov, O. Horovitz, M. Lipson, Ashdod, Israel
Interest ofDetection ofAntibodies in CT Positive and CT Negative Patients with
Genital Infection
Jeanne Orfila, C. Chaigneau, J.M. Sueur, Amiens, France
Clinical Research of Chlamydia trachomatis Infection in Women's Genitals
x,S, Wen, Y.J. Lin, G. Zhu P.R. China
4:20 4:50 GENERAL DISCUSSION AND SUMMARY
INFECTIOUSDISEASES IN OBSTETRICSAND GYNECOLOGY 115

				

